# The Role of *Nidogen* in Reproductive Fitness in *Drosophila*

**DOI:** 10.3390/biology14091192

**Published:** 2025-09-04

**Authors:** Uwe Töpfer, Anne Holz

**Affiliations:** 1 Institut fuer Allgemeine Zoologie und Entwicklungsbiologie, Fachbereich 08 Biologie und Chemie, Justus-Liebig-Universitaet Giessen, Heinrich-Buff-Ring 38, 35392 Giessen, Germany; uwe.toepfer@biologie.uni-marburg.de; 2 Department of Biology, Developmental Genetics, Philipps University, 35043 Marburg, Germany

**Keywords:** basement membrane, collagen, egg chamber, fertility, muscle sheet, ovary

## Abstract

The basement membrane (BM) is a specialized extracellular matrix that provides crucial structural support to tissues and organs. Mutations in BM genes can affect this structural support and are often associated with human diseases, including fibrosis, diabetes, and cancer, and are considered a hallmark of aging. However, how the BM maintains this support in organs with constant mechanical stress is largely unknown. In the *Drosophila* ovary, the BM provides the mechanical cues that are required for egg development. We discovered that the glycoprotein *Ndg* is important for maintaining organ integrity during egg production. Loss of *Ndg* results in a reduced number of progeny due to unfertilized eggs. Furthermore, we observed a decreased number of developing eggs and a premature death of egg chambers during development. Our results suggest that *Ndg* plays an important role in fertility.

## Simple Summary

The basement membrane (BM), a thin protein matrix in the extracellular space, provides vital information to organs to maintain their physiological functions. However, we do not know which of the proteins in this matrix are important in which organ and what happens in these organs when they are lost. Genes that encode BM components are highly conserved between *Drosophila* and humans. This makes the fruit fly an interesting genetic model system to study genes that might be important for reproductive biology and human health. The *Drosophila* ovary, the female’s reproductive organ, constantly produces eggs. *Nidogen* (*Ndg*), one of the main components of the BM, plays an important role in maintaining reproductive fitness. Loss of *Ndg* results in reduced egg production in aging flies, which can be associated with the death of egg precursors.

## 1. Introduction

The basement membrane (BM) is a specialized extracellular matrix (ECM) that plays a crucial role in tissue morphogenesis and homeostasis, maintaining the structural integrity and function of tissues [1,2,3]. BMs are mainly composed of Laminins and Collagen IV, which form separate networks, as well as further components that link these networks, like the proteoglycan Perlecan and the glycoprotein Nidogen (Ndg) [4]. Ndg plays a pivotal function in preserving tissue homeostasis and dynamics by integrating diverse components of the ECM and modulating their molecular architecture and mechanical stability [5,6,7,8].

The function of *Ndg* is evident in a variety of phenotypes observed in different species. In mice, the functional loss of one of the two *Ndg* genes neither results in lethality nor disrupts the formation of the BM. However, *Ndg1* mutant animals exhibit neurological phenotypes, including spontaneous seizures and loss of hind leg control [9,10,11]. Double mutants also exhibit no embryonic lethality or disturbance to the formation of the BM, but perinatal lethality occurs due to incorrect formation of the BM-associated ECM, which disturbs heart and lung development, as well as causes syndactyly [5,12]. Analyses of *nid-1* mutants in *C. elegans* are also vital and fertile and show no abnormalities in the structure of the BM. However, fertility is reduced [13]. The animals exhibit movement disorders and the orientation of the longitudinal nerves as well as the arrangement of the neuromuscular connections are disturbed [13,14,15,16]. Finally, loss of *nid1a* in *Danio rerio* results in a reduction in body length [17]. Similarly, neurological defects in *Drosophila*, such as movement and orientation disorders, could be explained by defects in the innervation of the peripheral nervous system (PNS) and the incorrect arrangement of cilia in neurons. In addition, a role of *Ndg* for the stability and permeability of the BM could be shown in larval visceral muscles [7]. Thus, the current understanding of the role of *Ndg* is that it strengthens the BM and ensures its barrier function. Consequently, loss of *Ndg* function results in malformation of various organs, disturbed growth, and altered behavior across different species. At a molecular level, the interaction between Ndg and integrins may play a role in cell attachment. This is evidenced by the binding of Ndg-1 to αvβ3 and α3β1 dimers [18,19], as well as by a study showing that Ndg-1 acts as a tumor suppressor by mediating the polarization of macrophages through interaction with αvβ3 integrin [20]. A previous study has proposed that a complex of integrin, Ndg, and laminin regulates the stem cell niche and epidermal maintenance [21]. Furthermore, *Ndg* has been associated with several diseases by altering adhesion and signaling pathways. For example, *Ndg-1* was identified as a novel biomarker in patients with acute myeloid leukemia [22], *Ndg-2* activated the Akt signaling pathway in patients with glioma [23], and overexpression of *Ndg-2* in a mouse model led to hepatosteatosis and atherosclerosis [24].

To study the function of *Ndg*, the *Drosophila* ovary is a well-suited model to study the mechanical functions of BM in vivo. The fact that *Ndg* null mutants are viable [7] and that only one gene encodes for *Ndg* [25] simplifies the analysis of *Ndg* function in *Drosophila*. The *Drosophila* ovaries are the paired reproductive organs of female flies and are composed of 16 ovarioles each. The germarium, located at the tip of these ovarioles and comprising the stem cell niche, produces egg chambers, the precursors of eggs. These are pushed towards the oviduct by a surrounding muscle sheet, where they are subsequently fertilised and laid. Finally, the entire ovary is surrounded and attached together with a thin muscle network called the peritoneal sheath [26,27]. Both, the germarium together with all egg chamber stages as well as the surrounding muscle sheets are surrounded by a Ndg-containing BM [6,28]. However, the molecular mechanisms by which *Ndg* maintains tissue integrity, and how loss of *Ndg* function can lead to behavioral disorders, reduced fertility, and human disease, are not yet fully understood.

Here, we identify *Ndg* as an important gene for maintaining fertility in female *Drosophila* flies. We discovered an unknown link between a BM component, age, and the number of offspring in the fruit fly. *Ndg* mutants exhibit accelerated reduced fertility in aging flies, which correlates with the residual level of Ndg protein in the respective mutant. We observed ovarioles with an empty muscle sheet towards the oviduct and premature apoptotic egg chambers in affected amorphic *Ndg* mutants. Together, these results suggest a role for *Ndg* in maintaining the integrity of the BM and the ovary’s ability to continuously produce eggs.

## 2. Materials and Methods

### 2.1. Fly Stocks and Genetics

The fly stocks used were *white^1118^* [Bloomington Drosophila Stock Center (BDSC), 3605, Bloomington, IN, USA] *Ndg^Δ0.4^*, and *Ndg^Δ1.4^* [7] (deletion generated from *Mi{ET1}MB04184*, *P{hsILMiT} and P{Δ2–3}99B)*, *Df Ndg* (BDSC_23666) [29], and *vkg::GFP* [Drosophila Genetic Resource Center (DGRC), 110626, Kyoto, Japan] [30]. Flies were kept at 25 °C on standard food [31]; *white^1118^* control flies were used because the *Ndg^Δ0.4^* and *Ndg^Δ1.4^* mutants used have the *white^1118^* mutant background.

### 2.2. Immunohistochemistry

Ovaries from flies with the indicated age were dissected in PBS and fixed with 4% formaldehyde in PBS. Rabbit anti-Nidogen (Ndg) antibody [32] was used with a dilution of 1:500 and rabbit anti-green fluorescent protein (GFP, Abcam, ab290, Cambridge, UK) was used with a dilution of 1:500. Alexa Cy-coupled secondary antibodies were purchased from Dianova, Hamburg, GER and Jackson ImmunoResearch, Cambridgeshire, UK and Hoechst 55,380 (1:1000, Sigma Aldrich, 94403, St. Louis, MO, USA) and rhodamine–phalloidin (1:200, Thermo Fisher Scientific, R415, Waltham, MA, USA). Embryos were embedded in Fluoromount-G (Southern Biotech, 0100-01, Birmingham, AL, USA) before visualization under a Leica TCS SP2 (Leica, Wetzlar, Germany) or Olympus FV1000 confocal microscope (Olympus, Tokyo, Japan). For comparison of Ndg protein levels in the controls and *Ndg* mutants, we used the same laser intensity and settings.

### 2.3. Fertility Test

In order to investigate the fertility of females, 15 virgin flies of the desired genotype were collected and mated with 5 young males of the control genotype *w^1118^* and kept at 25 °C in deposition vials. Flies were transferred to fresh vials every 24 h. Once the parents had reached the target age, the deposits were incubated at 25 °C for at least two hours to allow further development. The eggs obtained from the deposits were dechorionized, fixed, and de-vitellinized. The eggs were transferred directly to PBS with 0.1% Tween, stained with Hoechst, washed, and then analyzed. Eggs in which no DNA staining could be detected were interpreted as unfertilized.

### 2.4. Quantification of Egg Chamber Amount and Premature Apoptotic Egg Chambers

Flies were aged at 25 °C for 5 to 13 days, respectively, after which their ovaries were dissected in PBS. The ovarioles were loosened and fixed in 4% formaldehyde in PBS. The ovaries were stained with Hoechst, and the total number of egg chambers and the number of egg chambers per stage of oogenesis (grouped into stages 1–6, 7–12, and 13–14) were determined. Egg chamber stages of oogenesis were determined as previously described [26]. Premature apoptotic egg chambers were identified by condensed nuclei via the oversaturated fluorescence intensity of Hoechst staining. We counted egg chambers as prematurely apoptotic if apoptotic follicle or nurse cell nuclei appeared prior to stage 11 or 14, respectively, as these cells typically undergo apoptosis in these stages [33].

### 2.5. Statistical Analysis

The data were tested for equal variance and normal distribution, and statistical significance was subsequently calculated using a two-sided Student’s *t*-test for the comparison of two groups, and a Student’s *t*-test with Bonferroni correction for multiple comparisons, using R (version 2025.05.1+513) [34].

## 3. Results

### 3.1. Nidogen Is Required for Reproductive Fitness in Aging Flies

**Figure 1:**
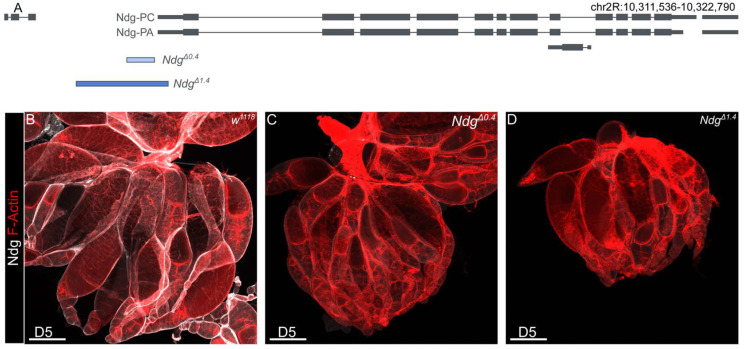

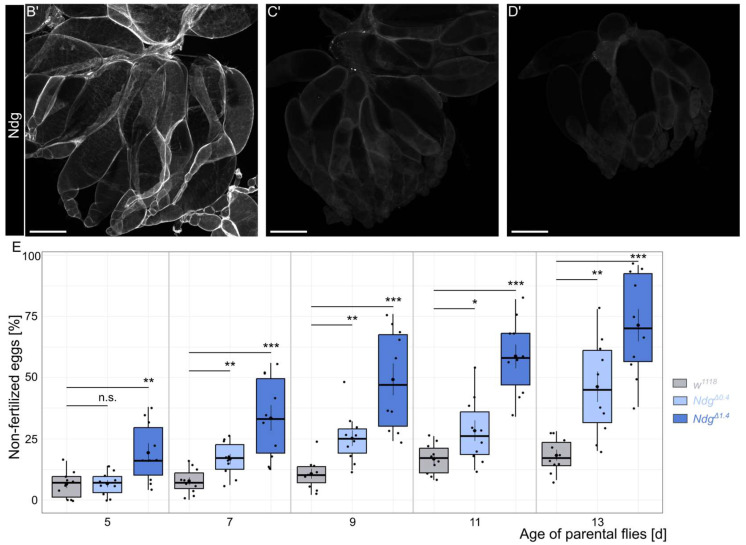
Role of *Nidogen* for *Drosophila* fertility. (**A**) Schematic overview of the *Nidogen* gene and deleted regions of *Ndg^Δ0.4^* (indicated in light blue) and *Ndg^Δ1.4^* (indicated in dark blue) mutants. (**B**–**D’**) Ovaries of *w^1118^* controls (**B**), *Ndg^Δ0.4^* (**C**), and *Ndg^Δ1.4^* (**D**) 5-day-old flies stained with anti-*Nidogen* antibody (white) and (**B**–**D**) for F-actin (red). Control ovaries show strong Ndg protein localization (**B**,**B’**), while *Ndg* mutants show apparent reduced *Ndg* signal for *Ndg^Δ0.4^* (**C**,**C**’) and abolished *Ndg* signal for *Ndg^Δ1.4^* (**D**,**D’**). Scale bars = 200 µm. (**E**) Boxplots showing the percentage of non-fertilized eggs of *w^1118^* controls (gray), *Ndg^Δ0.4^* (light blue), and *Ndg^Δ1.4^* (dark blue) genotypes laid by parental flies aged 5–13 days. Boxplots indicate the median, the 25th percentile, and the 75th percentile. Means (central black point); and individual measurements (smaller black points/scatterplot) are shown. * *p* < 0.025, ** *p* < 0.005, *** *p* < 0.0005 (Student’s *t*-test with Bonferroni correction). n.s. = not significant. n = 10 replicates of 50 eggs each.

*Ndg* is one of the major components of the BM. The loss of *Ndg* reveals roles in BM stability, barrier function, and nervous system patterning, although *Ndg* null mutants are viable [7]. To investigate a potential role of *Ndg* for *Drosophila* fertility, we used our previously generated *Ndg^Δ0.4^* and *Ndg^Δ1.4^* mutants (Figure 1A) [7]. Immunohistochemical analysis of *Drosophila* embryos using a specific Ndg antibody, as well as Western blot analysis, revealed that *Ndg^Δ0.4^* is a hypomorphic allele, while *Ndg^Δ1.4^* is amorphic. This is attributed to the fact that the hypomorphic allele shows residual expression, whereas the amorphic alleles do not [7,35]. To test if Ndg antibody staining of the ovary led to similar results, we repeated this experiment. We stained the ovaries of 5-day old *w^1118^* control flies as well as *Ndg^Δ0.4^* and *Ndg^Δ1.4^* mutants and co-stained the ovary with F-actin, which allows us to show muscles and discriminate between different stages of egg chamber development (Figure 1B–D’). In control flies, we observed prominently Ndg localization in BMs of the muscle sheets as well as of the epithelial follicle cells of the egg chambers (Figure 1B,B’). In *Ndg^Δ0.4^*, only a small amount of Ndg can be detected in the BM (Figure 1C,C’), while the specific Ndg signal was abolished in the *Ndg^Δ1.4^* mutants (Figure 1D,D’). *Ndg* is known to ensure proper fertility in *C. elegans* [13]. To test if the loss of *Ndg* affects female fertility, we quantified the percentage of non-fertilized eggs with progressive age over time from day 5 to day 13 (Figure 1E). During this period, the number of unfertilized eggs in the control group also began to increase slightly (Figure 1E). While in 5-day old flies, *Ndg^Δ1.4^* mutants showed a significantly higher percentage of non-fertilized eggs compared to controls (by ∼6.4% and ∼19.2%, respectively), while *Ndg^Δ0.4^* mutants did not (∼7%); furthermore, 7- to 13-day-old females showed significant differences between controls compared to both *Ndg* mutants alleles (listed in the order of controls, *Ndg^Δ0.4^*, and *Ndg^Δ1.4^*: ∼8%, ∼17%, and ∼33% for 7-day-old flies, ∼11%, ∼25%, and ∼49% for 9-day-old flies, ∼17%, ∼28%, and ∼59% for 11-day-old flies, ∼18%, ∼46%, and ∼71% for 13-day-old flies) (Figure 1E). Thus, a reduction in Ndg protein levels affects female fertility with higher effects in older flies.

### 3.2. Premature Apoptosis in Egg Chambers of Ndg Mutant Flies

**Figure 2:**
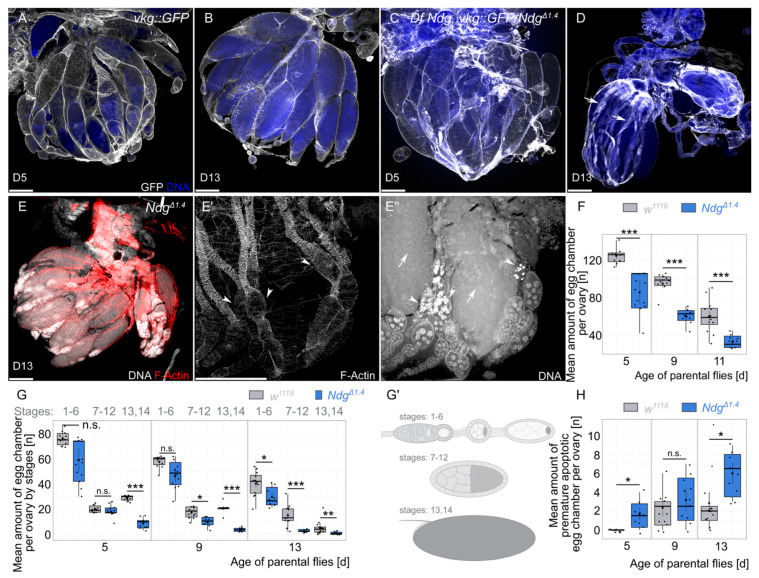
Morphological defects of *Ndg* mutants. (**A**–**D**) Anti-GFP antibody (white) and DNA (blue) staining on ovaries of 5-day-old (**A**,**C**) and 13-day-old (**B**,**D**) flies with the *vkg::GFP* control (**A**,**B**) and *Df Ndg*, *vkg::GFP/Ndg^Δ1.4^* (**C**,**D**) genotypes. (**E**–**E’’**) F-actin (red) and DNA (white) staining on *Ndg^Δ1.4^* mutants. In contrast to the controls (**B**), 13-day-old *Ndg* mutant conditions show partially empty ovarioles and highly condensed nuclei of mid-stage egg chambers (**D**–**E’’**). Scale bars = 200 µm. (**F**) Boxplots showing mean amount of egg chambers per ovary of *w^1118^* controls (gray) and *Ndg^Δ1.4^* (dark blue) genotypes of 5-, 9-, and 13-day-old flies. (**G**) Boxplots showing mean amount of egg chambers per ovary by stages of *w^1118^* controls (gray) and *Ndg^Δ1.4^* (dark blue) genotypes of 5-, 9-, and 13-day-old flies. (**G’**) Schematic illustration related to G, showing groups of stages 16, 7–12, and 13–14. (**H**) Boxplots showing mean amount of premature apoptotic egg chambers per ovary by stages of *w^1118^* controls (gray) and *Ndg^Δ1.4^* (dark blue) genotypes of 5-, 9- and 13-day old flies. Boxplots indicate the median, the 25th percentile, and the 75th percentile. Points indicate outliers below the 25th percentile or above the 75th percentile. Means (central black point); and individual measurements (smaller black points/scatterplot) are shown. * *p* < 0.05, ** *p* < 0.01, and *** *p* < 0.001 (Student’s *t*-test). n.s. = not significant. n = 12 ovaries of ≥6 flies.

To examine the reason for the lack of fertility of aging *Ndg* mutants, we studied the morphology of the ovaries using DNA and F-actin staining as well as the Collagen IV signal (Figure 2). We started to test if the Collagen IV signal is disturbed by visualizing Collagen IV with a GFP-tagged *vkg::GFP* fly line (*vkg* encodes the *Drosophila* Collagen IVa2 subunit). We tested the Collagen IV fluorescence signal in 5-day-old and 13-day-old controls as well as in a transheterozygous situation with the previously mentioned *Ndg^Δ1.4^* allele and a deficiency allele, where the *Ndg* locus is completely deleted [25], recombined with *vkg::GFP*. This strategy allows us on the one hand to study ovary morphology in a genetic null background and on the other hand it reduces the possibility of potential off-target phenotypes due to the independent *Ndg* alleles. While the Collagen IV signal is constantly high between different ages and genotypes (Figure 2A–D), *Ndg* mutant ovaries of 13-day-old flies are smaller and contain empty ovarioles that only show the BM of collapsed muscle sheets, missing the matured egg chambers within (Figure 2D, arrows). We next validated if we are able to observe similar phenotypes in the strong *Ndg^Δ1.4^* mutants and we found the same phenotype (Figure 2E–E’’). While some ovarioles are still filled with egg chambers of later stages (Figure 2E), parts of the ovarioles are only filled with early- and mid-staged egg chambers and are empty towards the oviduct (Figure 2E’,E’’, arrowheads). This partial lack of late egg chambers in the ovarioles is reflected by significant differences in the number of egg chambers in 5- to 13-day-old flies (∼125 and ∼85 at day 5, ∼95 and ∼60 at day 9, and ∼60 and ∼33 at day 13, respectively) (Figure 2F–G’). While follicle cell nuclei start to typically condense during stage 14 and nurse cell nuclei start to die from stage 11 on [33], we observed nuclei that condensate prematurely in earlier stages (Figure 2E’’, arrowhead, and Figure 2H). In particular, the presence of prematurely dying egg chambers correlates with ovarioles where no later egg chambers can be observed (compare Figure 2E’, and Figure 2E’’). In conclusion, in *Ndg* mutant ovaries, the number of egg chambers is reduced, especially that of later stages, and egg chambers prematurely die during development.

## 4. Discussion

The integrity of BMs is the key to the proper functioning of tissues and organs. The *Drosophila* ovary must sustain constant mechanical stress for ongoing egg production. The work described here provides insights into the role of *Ndg* to mediate the proper BM integrity. *Drosophila* encodes only one *Ndg* gene and in our *Ndg^Δ1.4^* mutant, Ndg protein is completely abolished (Figure 1D,D’) [7]. In *Ndg* mutants, the number of eggs produced per day is significantly reduced, which is an effect that increases with the aging of flies. In aged flies, we observed a reduced number of egg chambers present in the ovary; in particular, aged flies lacking late egg chamber stages in a part of the ovarioles. Finally, our data show that ovarioles without late egg chambers show prematurely dying egg chambers, which seem to get stuck in the ovariole.

Ndg is one of the most abundant proteins in the BM and has multiple binding partners including all major components, namely Laminin, Collagen IV, and Perlecan [36,37,38]. These multiple interactions have been shown to influence the mechanical properties of the BM. In particular, loss of Ndg have been shown to influence BM stiffness in the egg chamber [28], maintain BM topology in the wing disc [39], and modulate Collagen IV turnover during *Drosophila* embryonic development [40] as well as in *C. elegans* larval pharynx [41]. These effects occur at the molecular level and manifest as behavioral disorders, defective innervation, reduced body size, impaired lung and heart development, and syndactyly across different organisms [5,12,13,14,15,16,17]. Interestingly, similar to our results in *Drosophila*, in *C. elegans*, the fertility is also reduced, which has been determined by a reduced number of laid eggs [13]. However, in this study the number of eggs in the uterus was not significantly different between controls and *Ndg* mutants, which might be indicating that *Ndg* has distinct functions in *Drosophila* egg development. In the *Drosophila* visceral musculature, ultrastructural analyses revealed a perforated BM [7], which resulted in strong phenotypes, particularly in tissues under tension. Such defects could affect the physiology of the muscle sheet surrounding the ovary and impair fertility. The defects in the ovary could be due to mechanical stress, which the BM without *Ndg* may not be able to withstand adequately. Since BM stiffness not only affects migration but also the maintenance of tissue homeostasis and stem cell differentiation [42]. Thus, our results showing prematurely dying egg chambers might indicate a disturbance in the homeostasis of the stem cell niches in the ovary. BM components play an essential role in testes and ovaries in the positioning of the stem cell niche and thus for the maintenance of further differentiation processes [43,44,45,46]. It would be interesting to see if *Ndg* is also required for fertility in other organisms or if mutations in *Ndg* genes are a genetic condition associated with infertility in humans.

One thing we could not clarify in our study is the question: How is the premature dying of egg chamber related to the fertility defects? Dying egg chambers might stick in the muscle sheet that is required for its contraction to push forward developing egg chambers that are necessary for fertilization. Therefore, apoptotic egg chambers may interfere with egg activation and fertilization in the uterus. Egg activation depends on mechanical stress [47], which may be absent if BM integrity is compromised.

We identified a role of *Drosophila Ndg* for female reproductive fitness. Loss of *Ndg* reduces the number of laid eggs, with progressively stronger effects with age. Furthermore, in aged flies we observed fewer developing egg chambers and more prematurely dying egg chambers, which stuck in the ovarioles. Based on our findings and previous results in other species, we can now speculate that *Ndg* plays a conserved role in reproductive fitness across the animal kingdom. We hypothesize that *Ndg* is required in the BM to sustain the mechanical stress of the ovary during ongoing egg production and to maintain the microenvironment that regulates stem cell differentiation.

## 5. Conclusions

*Ndg* is one of the most highly conserved BM components, but its precise role is not yet fully understood. This study and others underline its role in maintaining tissue and organ integrity. However, further analyses are needed to explain the defects in terms of reduced fertility. Additionally, the roles of *Ndg* in barrier function and BM stability are also aspects that influence fitness. While *Ndg* itself may not be essential for viability, these results align with previous findings, providing a clearer picture of how *Ndg* can affect reproductive fitness.

## Data Availability

Data reported in this paper will be shared by the corresponding author upon request.

## Abbreviations

The following abbreviations are used in this manuscript: BM, basement membrane; DNA, deoxyribonucleic acid; F-actin, filamentous actin; GFP, green fluorescent protein; *Ndg*, *Nidogen*; PBS, phosphate buffer saline.
